# Clinical Outcomes of Image Guided Adaptive Hypofractionated Weekly Radiation Therapy for Bladder Cancer in Patients Unsuitable for Radical Treatment

**DOI:** 10.1016/j.ijrobp.2017.01.239

**Published:** 2017-05-01

**Authors:** Shaista Hafeez, Fiona McDonald, Susan Lalondrelle, Helen McNair, Karole Warren-Oseni, Kelly Jones, Victoria Harris, Helen Taylor, Vincent Khoo, Karen Thomas, Vibeke Hansen, David Dearnaley, Alan Horwich, Robert Huddart

**Affiliations:** ∗ The Institute of Cancer Research, London; † The Royal Marsden NHS Foundation Trust, Sutton, Surrey; ‡ The Royal Marsden NHS Foundation Trust, London

## Abstract

**Purpose and Objectives:**

We report on the clinical outcomes of a phase 2 study assessing image guided hypofractionated weekly radiation therapy in bladder cancer patients unsuitable for radical treatment.

**Methods and Materials:**

Fifty-five patients with T2-T4aNx-2M0-1 bladder cancer not suitable for cystectomy or daily radiation therapy treatment were recruited. A “plan of the day” radiation therapy approach was used, treating the whole (empty) bladder to 36 Gy in 6 weekly fractions. Acute toxicity was assessed weekly during radiation therapy, at 6 and 12 weeks using the Common Terminology Criteria for Adverse Events version 3.0. Late toxicity was assessed at 6 months and 12 months using Radiation Therapy Oncology Group grading. Cystoscopy was used to assess local control at 3 months. Cumulative incidence function was used to determine local progression at 1 at 2 years. Death without local progression was treated as a competing risk. Overall survival was estimated using the Kaplan-Meier method.

**Results:**

Median age was 86 years (range, 68-97 years). Eighty-seven percent of patients completed their prescribed course of radiation therapy. Genitourinary and gastrointestinal grade 3 acute toxicity was seen in 18% (10/55) and 4% (2/55) of patients, respectively. No grade 4 genitourinary or gastrointestinal toxicity was seen. Grade ≥3 late toxicity (any) at 6 and 12 months was seen in 6.5% (2/31) and 4.3% (1/23) of patients, respectively. Local control after radiation therapy was 92% of assessed patients (60% total population). Cumulative incidence of local progression at 1 year and 2 years for all patients was 7% (95% confidence interval [CI] 2%-17%) and 17% (95% CI 8%-29%), respectively. Overall survival at 1 year was 63% (95% CI 48%-74%).

**Conclusion:**

Hypofractionated radiation therapy delivered weekly with a plan of the day approach offers good local control with acceptable toxicity in a patient population not suitable for radical bladder treatment.

**Summary**

This prospective study demonstrates that hypofractionated radiation therapy delivered with a plan of the day approach is well tolerated in patients unfit for radical bladder cancer treatment. It provides opportunity for local disease and symptom control in patients for whom cystectomy, trimodality, or daily radiation therapy is not appropriate.

## Introduction

The underuse of curative therapy in patients with muscle invasive bladder cancer (MIBC) is well documented 1, 2, 3. High cancer-specific mortality is particularly evident in older patients, reflecting their poorer access to effective treatment (2).

There is good evidence that symptomatic local disease can be relieved for the duration of survival with hypofractionated radiation therapy (21 Gy in 3 fractions on alternate days) when either cystectomy or radical radiation therapy is unsuitable (4). Inasmuch as local disease control is related to total radiation therapy dose delivered, a higher biological effective dose is anticipated to improve outcomes 5, 6. Several retrospective studies report successful treatment of MIBC with 30 to 36 Gy in 6 weekly fractions 7, 8, 9, 10. Each fraction of this regimen represents ∼17% of the prescription dose, so a geographic miss could potentially compromise tumor control and tolerability.

Image guided adaptive radiation therapy (IGRT) strategies in bladder cancer show significant dosimetric gains for tumor coverage and normal tissue sparing 11, 12, 13, 14, 15. Implementation is therefore anticipated to improve local control and toxicity, but prospective studies are lacking. Here we report on the long-term clinical outcomes of “plan of the day” hypofractionated radiation therapy in a nonrandomized phase 2 study in patients unsuitable for radical treatment.

## Methods and Materials

Between January 2009 and March 2014, 55 patients with pathologic evidence of MIBC who were unsuitable for cystectomy or daily radiation therapy because of stage, comorbidity, or personal preference were recruited prospectively to an institutional Clinical Research and Ethics Committee approved protocol (NCT01000129; ISRCTN80815524).

Details of planning and plan selection have been previously presented and are summarized in Table E1 (available online at www.redjournal.org) 12, 16. Normal tissue constraints are given in Table E2 (available online at www.redjournal.org). Treatment was delivered using plan selection from a library of three 3-dimensional (3D) conformal plans treating whole empty bladder to 36 Gy in 6 fractions over 6 weeks.

Acute toxicity was collected using the Common Terminology Criteria for Adverse Events version 3.0 at baseline, weekly during radiation therapy, and then at 6 and 12 weeks after the completion of treatment. Local response was assessed at 3 months with cystoscopy and biopsy where possible. Local response was defined as absence of pathologic or clinical MIBC at cystoscopy, pathologic or radiologic downstaging to noninvasive disease, or stable radiologic disease.

Late toxicity was scored at 6 months and 12 months using the Radiation Therapy Oncology Group (RTOG) late radiation morbidity-scoring schema. Patients were subsequently followed up as clinically indicated, with cystoscopic surveillance and imaging where appropriate.

Overall survival was estimated using the Kaplan-Meier method and was defined as time from start of radiation therapy to death resulting from any cause. Surviving patients and those lost to follow-up were censored at the last assessment date. Cumulative incidence competing risk (CICR) method was used to calculate local progression at 1 and 2 years. Local progression was defined as interval from the start of radiation therapy to disease relapse within the bladder (pathologic, radiologic, or clinical). Death without local progression was treated as a competing risk. CICR analysis was performed using R version 3.3.1. All other analyses were carried out using STATA v13.1 (StataCorp LP, TX).

### Statistical considerations

Sample size was calculated assuming the rate of local control was no more than 40% at 3 months (null hypothesis). Using a single-stage design with 1-sided α of 0.05 (assuming true rate of control is 60%), 56 patients were estimated for 90% power. Considering expected loss to follow-up before the 3-month evaluation, recruitment target was set at 67 patients. The study closed before this because of a competing study (HYBRID study; NCT01810757) (17).

## Results

Patient characteristics are shown in Table 1. Target coverage and normal tissue sparing have been previously presented (12). Seven patients stopped treatment early because of deterioration in their general health. Four deaths occurred during radiation therapy unrelated to treatment (3 from pneumonia and 1 from urinary sepsis).

### Treatment-related toxicity

Radiation therapy was well tolerated, with no grade 4 genitourinary or gastrointestinal toxicity seen at any time.

**Fig. 1:**
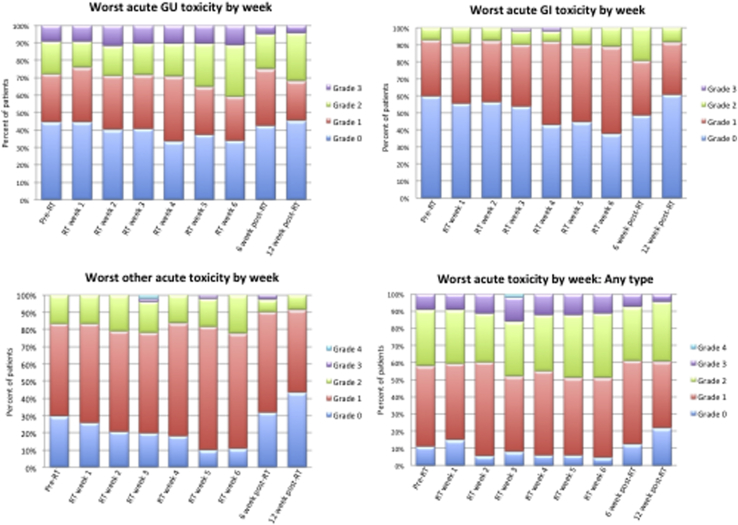
Worst symptoms or acute toxicity as graded by the Common Terminology Criteria for Adverse Events CTCAE version 3.0. Total number of patients available for assessment at each time point experiencing any toxicity: before radiation therapy (RT), 55; RT week 1, 54; week 2, 53; week 4, 49; week 5, 49; week 6, 45; 6 weeks after RT, 41; 12 weeks after RT, 23. *Abbreviations:* GI = gastrointestinal; GU = genitourinary.

At baseline (before radiation therapy), grade 2 and grade 3 urinary toxicity was evident in 10 (19%) and 5 (9%) patients. During treatment, acute genitourinary grade 2 and grade 3 toxicity was seen in 22 (40%) and 10 (18%) patients, respectively (Fig. 1). At 6 weeks after radiation therapy, 8 patients (20% of those assessed) had grade 2 urinary toxicity, and 2 patients (5% of those assessed) had grade 3 urinary toxicity. At 12 weeks after radiation therapy 6 patients (27% of those assessed) reported grade 2 toxicity, and 1 patient (4.5% of those assessed) had grade 3 urinary toxicity.

Acute gastrointestinal grade 2 and grade 3 toxicity was seen in 21 (38%) and 2 (4%) patients, respectively. Change in acute toxicity over time is shown in Figure E1 (available online at www.redjournal.org). Figure E2 (available online at www.redjournal.org) shows total number of patients available for assessment at each time point.

Other acute grade 2 toxicity occurred in 23 (42%) patients (predominately fatigue or anemia). Other acute grade 3 toxicity occurred in 2 (4%) patients (hyponatremia and syncope). One grade acute 4 toxicity event occurred (ventricular arrhythmia), unrelated to radiation therapy. No known radiation therapy treatment-related deaths occurred during the follow-up period.

**Fig. 2:**
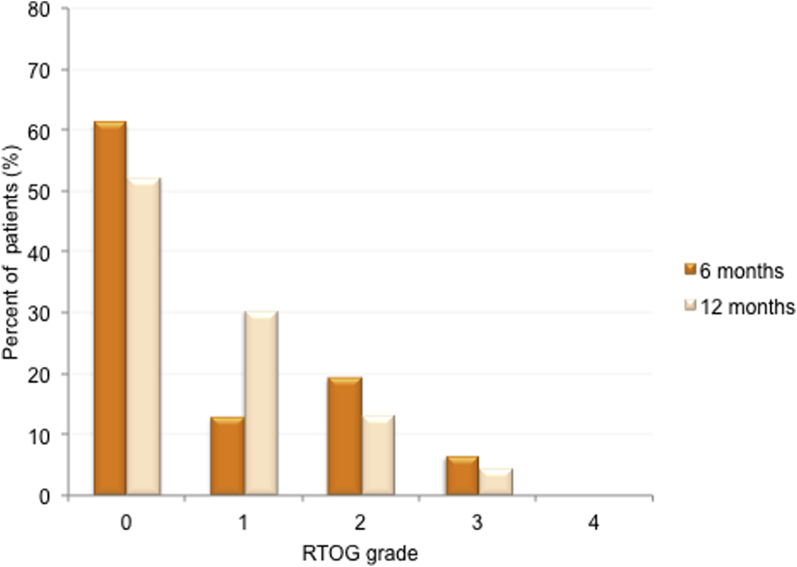
Worst late toxicity at 6 and 12 months as graded by Radiation Therapy Oncology Group (RTOG). Total number of patients available for assessment at each time point experiencing late toxicity: 6 months, 31; 12 months, 23.

The RTOG late toxicity scores at 6 and 12 months were available for 31 and 23 patients, respectively. Grade 2 late toxicity of any type at 6 and 12 months was seen in 6/31 (19%) and 3/23 (13%) patients, respectively. Grade 3 late toxicity of any type at 6 and 12 months was seen in 2/31 (6.5%) and 1/23 (4.3%) patients, respectively (Fig. 2). At 6 months, 5 patients (16.1%) reported grade 2 and 2 patients (6.5%) reported grade 3 RTOG bladder toxicity. At 12 months, 2 patients (8.7%) reported grade 2 and 1 patient (4.3%) reported grade 3 RTOG bladder toxicity. All late bladder symptom scoring (grade 2 and grade 3) were as a result of cystitis-like symptoms (frequency, urgency, and dysuria). No episodes of ≥grade 2 hematuria were reported at 6 or 12 months.

No ≥3 grade late bowel toxicity was seen. Two patients experienced grade 2 toxicity at 6 months (6.5%), and 1 patient (4.3%) had grade 2 toxicity at 12 months. All late RTOG bowel symptoms (scoring at grade 2) were as a result of diarrhea.

### Response assessment and outcome

**Fig. 3:**
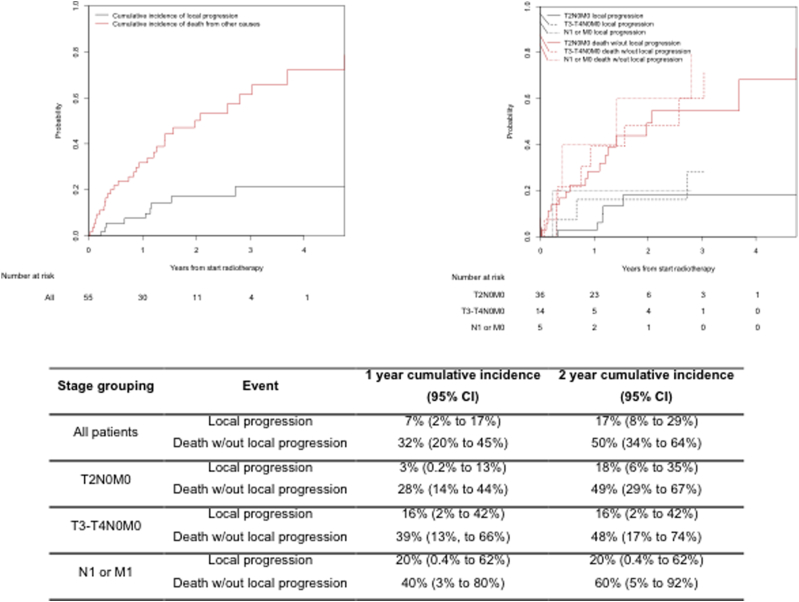
Cumulative incidence of local progression using death resulting from other causes as competing risk, for all patients and stratified by stage. *Abbreviation:* CI = confidence interval.

Response to radiation therapy 3 months after completing treatment was assessed in 36 patients. This was performed by cystoscopy in 30 patients. Thirty-three of 36 (92%) of assessed patients (60% of all patients) achieved local disease control within the bladder at 3 months, and 28/30 (93%) achieved complete response as assessed by cystoscopy. This means that local control at 3 months was achieved in a minimum of 51% (28/55) of all patients (based on assuming failure in all patients not assessed by cystoscopy). Cumulative incidence of local progression was 7% (95% confidence interval [CI] 2%-17%) and 17% (95% CI 8%-29%) for all patients at 1 year and 2 years, respectively. Results for patients stratified by stage are presented in Figure 3.

**Fig. 4:**
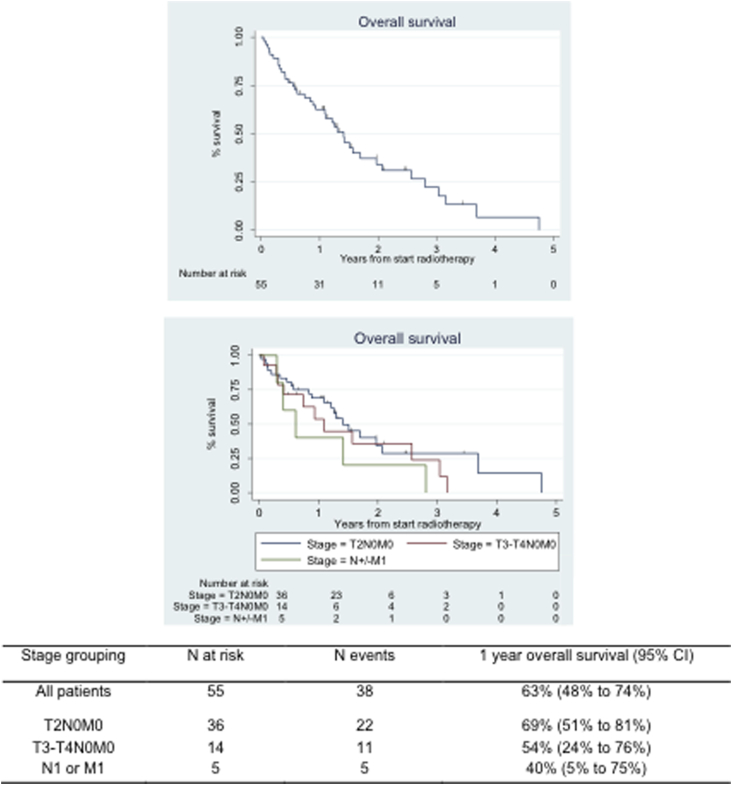
Kaplan-Meier plots for rates of overall survival and stratified by stage. *Abbreviation:* CI = confidence interval.

After a median follow-up time of 2 years, a total of 38 deaths were recorded. Estimated survival at 1 year was 62% (95% CI 48%-74%) (Fig. 4). Table 2 summarizes the outcomes grouped by stage.

## Discussion

Our study demonstrates that patients with high median age and comorbidity index who are deemed unsuitable for radical treatment can be treated with hypofractionated weekly radiation therapy delivered with a plan of the day approach with acceptable morbidity rates.

Given cancer-specific mortality is highest in individuals older than 80 years, the assumption that death resulting from competing medical conditions rather than from MIBC justifies no active treatment does not necessarily hold true (2). Intervention (cystectomy, radiation therapy, or transurethral resection alone) for older patients significantly reduces risk of death from MIBC compared with watchful waiting 2, 3. Watchful waiting alone, we contend, also would have been suboptimal management, given that 85% of our patient population had potentially curable disease (T2 or T3 only disease). Our data also support intervention for this population, as long-term survival and progression-free survival was achieved in a third of patients.

From the linear quadratic equation, 36 Gy in 6 fractions is approximately 48 Gy, assuming α/β of 10 for tumor control when delivered in 2 Gy per fraction (EQD2). It delivers a higher total dose than other commonly used radiation therapy regimens in the nonradical setting. The EQD2 of 20 Gy in 5 fractions, 21 Gy in 3 fractions, and 30 Gy in 10 fractions are 23 Gy, 30 Gy, and 33 Gy, respectively. Although fewer fractions are less burdensome, to achieve balance between efficacy, toxicity, and convenience, patients' prescriptions should be individualized.

Turgeon et al (18) delivered 50 Gy in 20 fractions with non-IGRT intensity modulated radiation therapy, achieving EQD2 of 52 Gy. Their patients (median age, 70) were also suitable for concurrent chemotherapy (gemcitabine), which clinically improves local control and from radiobiological modeling has equivalent additional >10 Gy benefit (19). They achieved 83% complete response, 43% of which had assessment with cystoscopy and biopsy.

Biopsy assessment was not mandated within our study because that would have necessitated rigid cystoscopy and the use of anesthetic agents in a high-risk population. The absence of pathologic correlation (biopsy at the time of cystoscopy) lends itself to tumor understaging; however, any downstaging achieved (ie, partial response) has potential patient benefit (20).

Even though most patients (72%) had no significant urinary symptoms (grade 0-1) before receiving radiation therapy, urinary symptom scores did not deteriorate during radiation therapy, as may have been anticipated, given improved targeting with the plan of the day approach (12). There is some suggestion of possible improvement in scores after radiation therapy than at baseline, reflecting potential early symptom control.

Given that 1-year survival is 62% (95% CI 48%-74%); the risk of late toxicity is an important consideration, especially in the context of the large fraction size used, but the observed rate of late toxicity (≥grade 2) was low. The dosimetric advantage with plan of the day is likely to have contributed to both the high complete response and the low nonurinary toxicity rates (12). We acknowledge that owing to the nature of the trial population, a proportion of patients did not contribute toxicity information. Such missing data are inevitable, and illustrates the difficulties of assessing a less fit population.

A weakness of this work is that it is a single-center single-arm study. We shall address this within a randomized multicenter trial (HYBRID study; hypofractionated bladder radiation therapy with or without image guided adaptive planning NCT01810757) (17). Unlike the reported phase 2 study, this trial will allow treatment delivery using volumetric intensity modulated arc therapy. In addition to improving conformity, it will shorten treatment delivery time, thereby reducing the opportunity for intrafraction organ motion and filling (21). Further opportunity to spare normal tissue is likely to come from online reoptimization strategies in development (22).

In conclusion, hypofractionated bladder radiation therapy of 36 Gy in 6 weekly fractions delivered with image guidance presents an important disease control option in appropriately selected patients and meets the clinical need for a somewhat neglected patient population. Exclusion from radical treatment on the basis of age alone would be unacceptable because functional decline is specific to the individual. This approach is advocated not as an alternative to radical treatment but as a means of potential disease control in patients with competing comorbidities.

## Figures and Tables

**Table 1:** Patient characteristics

Characteristic	n
Age	Median, 86 years (range, 68-97)
Sex	
Male	31
Female	24
Stage at presentation∗	
T2N0M0	36
T3N0M0	11
T4N0M0	3
T_any_N1-N3M0	3
T_any_N_any_M1	2
Histologic subtype	
Transitional cell carcinoma†	49
Squamous cell carcinoma	3
Adenocarcinoma	1
Other	2
Grade	
Intermediate (grade 2)	1
High (grade 3)	52
Unknown	2
WHO performance status at baseline‡	
0	4
1	16
2	12
3	6
Unknown	17
Charlson comorbidity index (age adjusted)§	
0	21 (0)
1	11 (0)
2	12 (0)
3	3 (2)
4	5 (18)
≥5	3 (35)
Number of fractions delivered‖	
≤4	4
5	6
6	45
Assessment of response 3 months after radiation therapy	
Cystoscopy	30
Radiology alone	6
Not assessed	19

*Abbreviation:* WHO = World Health Organization.

∗ All patients were staged no more than 6 weeks before receiving radiation therapy according to the American Joint Committee on Cancer (7th edition) with computed tomography of the chest, abdomen, and pelvis. After November 2011, protocol amendment was approved to exclude those patients with nodal or metastatic disease.

† Includes variant transitional cell carcinomas.

‡ No patient had performance status >3 at baseline assessment; 17 patients did not have performance status documented at baseline assessment but were deemed fit for proposed treatment by recruiting clinician (ie, presumed performance status 0-3).

§ Modified Charlson-Deyo score (measure of comorbidity across multiple organ sites, captured using International Classification of Diseases, 9th revision, Clinical Modification codes) was retrospectively calculated for each patient excluding his or her diagnosis of bladder cancer at presentation. Age-adjusted Charlson index score: for each decade after 40 years an additional point is added.

‖ Three patients were planned to 30 Gy in 5 fractions because of either advanced disease or limited performance status; 48 patients completed their prescribed course of radiation therapy.

**Table 2:** Status at last follow-up visit after median follow-up time of 2 years

Status	Number of patients			Total patient number (%)
	Initial T2N0M0	Initial T3-T4N0M0	Initial TanyN + M1	
Alive	12	5	0	17 (30.9)
Disease free	10	5	0	15 (27.3)
Localized disease (bladder)	1	0	0	1 (1.8)
Regional disease (pelvis)	0	0	0	0 (0)
Metastases	1	0	0	1 (1.8)
Dead	24	9	5	38 (69.0)
Metastases	10	4	3	17 (30.9)
Other malignancy	1	1	0	2 (3.6)
Other causes∗	13	4	2	19 (34.5)

∗ No known deaths resulting from radiation therapy occurred; 19 other causes of death were pneumonia (8 patients), cardiac events (4 patients), urinary sepsis (2 patients), unknown cause of death (2 patients), sepsis from idiopathic myelosuppression and bowel obstruction (1 patient), peripheral vascular disease (1 patient), superior vena cava obstruction (1 patient).

## Supplementary Data

Tables E1 and E2 and Figures E1 and E2
