# Erratum to: Hafeez S, McDonald F, Lalondrelle S, et al. Clinical outcomes of image guided adaptive hypofractionated weekly radiation therapy for bladder cancer in patients unsuitable for radical treatment. *Int J Radiat Oncol Biol Phys* 2017;98:115-122.

**DOI:** 10.1016/j.ijrobp.2017.11.005

**Published:** 2018-02-01

The authors wish to bring to the readers' attention typographical error identified in Supplementary Table 2 with the online version of the article showing the dose constraints guidance used for 3D conformal planning for total prescription dose of 36Gy in 6 fractions (1). The content of the columns for other bowel were inadvertently moved in formatting. This was not recognized by the authors at the time of manuscript review. The corrected Supplementary Table 2 is as below. We apologize for any inconvenience.

**Supplementary Table 2:** Dose constraints guidance used for 3D conformal planning for total prescription dose of 36Gy in 6 fractions

Organ	Constraint		
Rectum (including anus)	17Gy	80%	
	28Gy	60%	
	33Gy	50%	
	36Gy	30%	
Femoral heads	28Gy	50%	
Other bowel (including small and large bowel as a single structure)		optimal	mandatory
	V25	139cc	208cc
	V28	122cc	183cc
	V31	105cc	157cc
	V33	84cc	126cc
	V36	26cc	39cc

Proposed constraints are based on total prescription dose of 36Gy prescribed to 100% at the International Commission on Radiation Units and Measurements reference point. In those with advanced disease or limited performance status 30Gy in 5 fractions was considered (three patients planned to 30Gy in 5 fractions). Dose constraints were derived from previously recruited phase III studies (CHHIP and BC2001) using linear quadratic model assuming α/β of 10 for tumor control and 3 for normal tissue 2, 3, 4, 5. Organs at risk were contoured as solid structures by defining their outer wall on CT0. Other bowel constraints were specified only for the small plan and medium plan as it was expected that the large plan would exceed above constraints given the position of bowel on the planning CT scan is not reflective of true bowel position at treatment delivery when large plan would be selected for treatment.
