# Supplementary material for: Clinical Outcomes of Image Guided Adaptive Hypofractionated Weekly Radiation Therapy for Bladder Cancer in Patients Unsuitable for Radical Treatment
Source: Int J Radiat Oncol Biol Phys. 2017 May 1;98(1):115–22. doi: 10.1016/j.ijrobp.2017.01.239 (PMC5392498; doi:10.1016/j.ijrobp.2017.01.239)

Supplementary Table 1. CTV to PTV expansion details

CT data set	PTV	CTV to PTV Expansion (cm)				
		Laterally	Anteriorly	Posteriorly	Superiorly	Inferiorly
CT0	PTV Small	0.5	0.5	0.5	0.5	0.5
CT0	PTV Medium	0.5	1.5	1.0	1.5	0.5
**If CTV on CT0 and CT30 <50 cm^3**						
CT0	PTV Large	0.75	2.0	1.2	2.5	0.75
**If CTV on CT0 and CT30 >50 cm^3**						
CT30	PTV Large	0.5	1.5	1.0	1.5	0.5

CT0, planning scan acquired immediately post voiding. CT30, planning scan acquired 30 minutes later with no drinking or voiding permitted between the two scans. CTV was contoured to encompass the visible tumour, whole bladder and any areas of extravesical spread. To model patient-specific filling, the PTV large margin was informed by the magnitude of bladder filling between CT0 and CT30. Pre-treatment CBCT was acquired and co-registered to the reference image (CT0) using automated bone match. Two trained radiographers selected the smallest PTV and corresponding plan that provided appropriate coverage of the bladder.

Supplementary Table 2. Dose constraints guidance used for 3D conformal planning for total prescription dose of 36Gy in 6 fractions

Organ	Constraint		
**Rectum** (including anus)	17Gy	80%	
	28Gy	60%	
	33Gy	50%	
	36Gy	30%	
**Femoral heads**	28Gy	50%	
**Other bowel** (including small and large bowel as a single structure)		optimal	mandatory
	V25	105cc	139cc
	V28	84cc	122cc
	V31	26cc	157cc
	V33	208cc	126cc
	V36	183cc	39cc

Proposed constraints are based on total prescription dose of 36Gy prescribed to 100% at the International Commission on Radiation Units and Measurements reference point. In those with advanced disease or limited performance status 30Gy in 5 fractions was considered (three patients planned to 30Gy in 5 fractions). Dose constraints were derived from previously recruited phase III studies (CHHIP and BC2001) using linear quadratic model assuming α/β of 10 for tumor control and 3 for normal tissue [1-4]. Organs at risk were contoured as solid structures by defining their outer wall on CT0. Other bowel constraints were specified only for the small plan and medium plan as it was expected that the large plan would exceed above constraints given the position of bowel on the planning CT scan is not reflective of true bowel position at treatment delivery when large plan would be selected for treatment.

References

1. James ND, Hussain SA, Hall E, Jenkins P, Tremlett J, Rawlings C, Crundwell M, Sizer B, Sreenivasan T, Hendron C *et al*: **Radiotherapy with or without chemotherapy in muscle-invasive bladder cancer**. *N Engl J Med* 2012, **366**(16):1477-1488.
2. Dearnaley D, Syndikus I, Sumo G, Bidmead M, Bloomfield D, Clark C, Gao A, Hassan S, Horwich A, Huddart R *et al*: **Conventional versus hypofractionated high-dose intensity-modulated radiotherapy for prostate cancer: preliminary safety results from the CHHiP randomised controlled trial**. *Lancet Oncol* 2012, **13**(1):43-54.
3. Pos FJ, Hart G, Schneider C, Sminia P: **Radical radiotherapy for invasive bladder cancer: What dose and fractionation schedule to choose?** *Int J Radiat Oncol Biol Phys* 2006, **64**(4):1168-1173.
4. McDonald F, Waters R, Gulliford S, Hall E, James N, Huddart RA: **Defining bowel dose volume constraints for bladder radiotherapy treatment planning**. *Clin Oncol (R Coll Radiol)* 2015, **27**(1):22-29.

Supplementary Figure 1. Change in acute toxicity over time

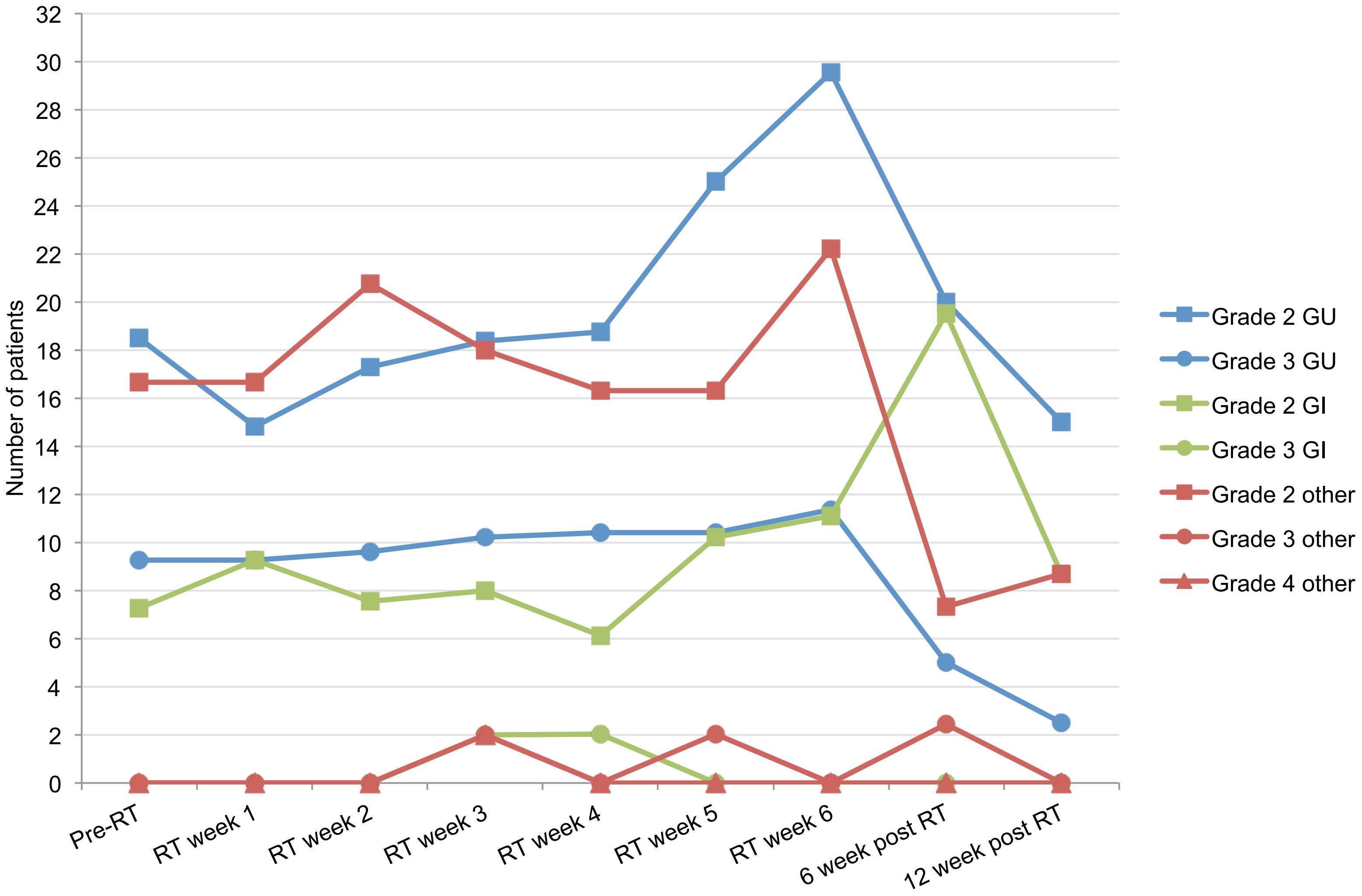

Total number of patients available for assessment at each time point is shown in Supplementary Figure 2

Supplementary Figure 2. Number of patients assessed at each time point

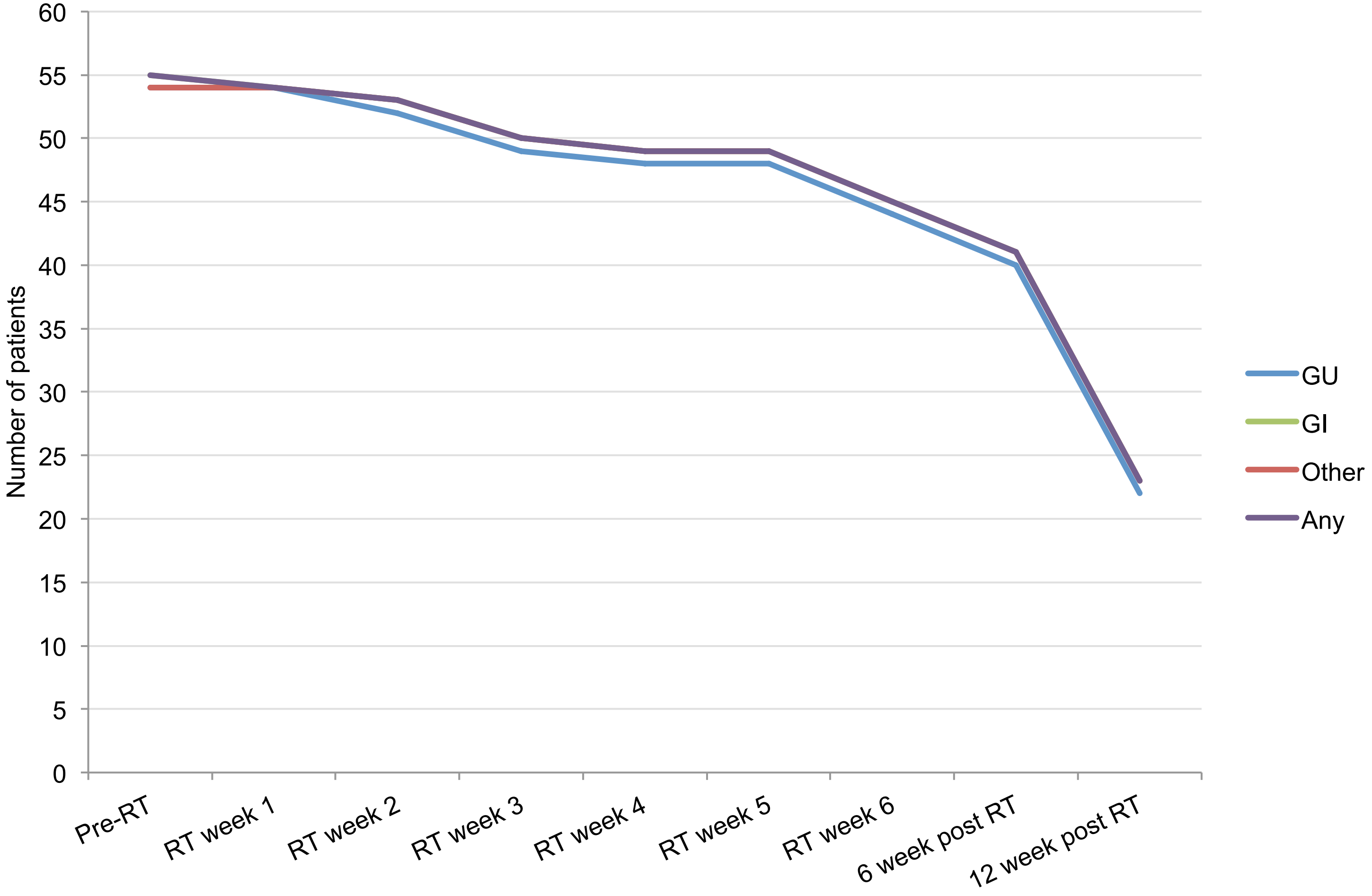

Supplement: Tables E1 and E2 and Figures E1 and E2 [file mmc1.pdf]
